# Heterogeneity in Short Video Addiction and Its Association with Inattention and Negative Emotions Among College Students

**DOI:** 10.3390/healthcare14050559

**Published:** 2026-02-24

**Authors:** Wei Zhao, Wenting Zhang, Shanshan Ma, Yuxuan Zhang, Yiping Nan, Xiaowei Li, Chengxu Duan, Shang Gao, Yangyi Zhou, Ying Zhang

**Affiliations:** 1 School of Nursing, Xi’an Jiaotong University Health Science Center, Xi’an 710061, China; 3124315116@stu.xjtu.edu.cn (W.Z.); 18809543992@stu.xjtu.edu.cn (W.Z.); mss@stu.xjtu.edu.cn (S.M.); galaxyexpress999@stu.xjtu.edu.cn (C.D.); gaoshang2002@stu.xjtu.edu.cn (S.G.); 3285403807@stu.xjtu.edu.cn (Y.Z.); 2 School of Nursing, Chinese Academy of Medical Sciences & Peking Union Medical College, Beijing 100144, China; s2024025028@student.pumc.edu.cn; 3 School of Nursing and Rehabilitation, Xi’an Medical University, Xi’an 710021, China; nanyiping@xiyi.edu.cn; 4 School of Nursing, Shaanxi University of Chinese Medicine, Xianyang 712046, China; 2041020@sntcm.edu.cn

**Keywords:** short video addiction, negative emotions, attention, college students, latent profile analysis

## Abstract

**Background/Objectives:** Short video addiction (SVA) has become a matter of public health concern, impacting the well-being of college students. However, previous studies have largely treated SVA as a homogeneous phenomenon, overlooking the potential heterogeneity in addictive behaviors among individuals and their underlying mechanisms. This study aims to identify distinct SVA subtypes and explore the mediating role of inattention (IA) in the relationship between these subtypes and negative emotions. **Methods:** The present study recruited a sample of college students through a multicentre online survey conducted from January to August 2025. Latent profile analysis identified distinct SVA categories among college students, and further examination of the mediating role of IA between SVA and negative emotions (anxiety, depression) was undertaken. **Results:** Latent profile analysis stratified SVA into three cohorts: “Healthy short video use” (8.9%), “Short video dependence” (55.8%), and “Short video addiction” (35.3%). The subtypes differed in levels of IA, anxiety, and depression (*p* < 0.05), with IA functioning as the link between SVA and negative emotions. It is noteworthy that IA demonstrated a complete mediating effect in the anxiety model, thereby indicating that the adverse impact of SVA on mental health is predominantly mediated by impaired attention function. **Conclusions:** This study deepens the understanding of students’ SVA from a heterogeneity perspective and provides empirical evidence for exploring the potential cognitive mechanisms through which SVA influences mental health. It is suggested that targeting attention function may hold great value in alleviating SVA-related psychological issues.

## 1. Introduction

The advent of Internet technology has precipitated a paradigm shift in the manner in which individuals engage with society, seek leisure, and entertainment. The proliferation of short videos as a nascent form of social media is indicative of this transformation [1]. A short video is defined as a type of online video with a duration ranging from a few seconds to a few minutes [2]. In comparison with traditional video media, this particular application is characterized by its emphasis on high-intensity stimulation, fragmented content, and algorithmic recommendations [2,3]. This makes it highly suitable for the demands of modern life, where efficiency in information acquisition is a key priority. User engagement with short-form video content is growing exponentially around the world. Platforms like TikTok and YouTube Shorts are reshaping people’s digital consumption patterns [4,5,6]. According to the 56th Statistical Report on Internet Development in China [7], as of June 2025, the number of Internet users had reached 1.123 billion. Furthermore, the number of short video users had reached 1.068 billion, with a user penetration rate of 95.1%. The ubiquity of short video applications has given rise to concerns regarding short video addiction (SVA), which has emerged as a significant issue. SVA is a type of behavioral addiction derived from the concept of Internet addiction and mobile social application addiction [8]. The term “SVA” is employed to denote a chronic or periodic obsessive state that arises from the repeated utilization of short video applications such as Douyin (TikTok) [9]. This condition is accompanied by a robust and persistent craving for the short videos, as well as a concomitant dependence. College students, as the primary users of Internet applications, constitute both the primary consumers of short videos and a high-risk group with regard to addictive behaviors [10]. This vulnerability is particularly pronounced among Generation Z, who are closely tied to their “mobile-first” lifestyle. As recent studies emphasize, digital self-efficacy and social influence are core drivers of Generation Z consumers’ technological engagement [11]. This social-oriented decision-making framework, in conjunction with their high digital literacy, may increase their vulnerability to SVA. Research indicates that over 80% of Chinese college students frequently watch short videos, with a significant proportion spending more than an hour daily on these platforms [12]. This high-frequency usage has given rise to concerns regarding SVA, with its prevalence estimated to range between 21.63% and 31.99% [13]. This addictive behavior has been demonstrated to have a detrimental effect on the academic performance of college students [14]. Furthermore, it has been shown to have a significant impact on the mental health of students, frequently giving rise to negative emotions such as depression and anxiety [8,15]. Furthermore, prolonged and frequent viewing of short videos has been shown to be closely associated with inattention (IA) [16,17]. Attention deficit has been demonstrated to have a detrimental effect on learning efficiency and academic achievement [18,19], and further exacerbates individuals’ negative emotions [20]. Nevertheless, the mechanisms underpinning the association between addiction and emotional distress remain to be elucidated. The present study aims to investigate the impact of SVA on negative emotions among college students, with a specific focus on the mediating role of attention deficit in this relationship.

Negative emotions are defined as unpleasant experiences felt by individuals, usually as a result of unfavorable events or interactions, and are often manifested as anxiety and depression, among others [21]. While short videos can alleviate stress and regulate emotions in the short term [22], prolonged excessive viewing may weaken an individual’s emotional regulation abilities, leading to heightened emotional fluctuations [23]. Emotion regulation theory posits that addictive behavior can be conceptualized as a non-adaptive strategy employed in the management of persistent negative emotions [24]. In instances where an individual’s capacity for internal emotion regulation is found to be inadequate, they are likely to seek external sources of stimulation in order to achieve transient emotional relief. Short videos enable individuals to reduce negative emotional feelings in the short term by personalizing content with immediate sensory stimulation [25]. However, long-term reliance on such external stimuli has the potential to diminish an individual’s emotional regulation ability, resulting in a rebound of negative emotions and an increase in dependence [26]. Numerous studies have shown a significant association between SVA and negative emotions. A substantial body of research has established a positive correlation between excessive use of social media, specifically short videos, and adverse emotional states, including anxiety and depression [27,28]. Compared to ordinary users, short video addicts scored significantly higher across multiple dimensions, including depression, anxiety, stress, and loneliness [29]. These studies collectively indicate that SVA has become a key risk factor for inducing negative emotions among college students.

IA is a significant cognitive feature of SVA. Short video platforms utilize a fast-paced, fragmented presentation model that is characterized by intense sensory stimulation and instant gratification. This rapid content turnover has been demonstrated to compel users to shift their attention frequently within short intervals. Over time, this behavioral pattern has a detrimental effect on an individual’s ability to sustain focus [30]. Empirical evidence indicates that individuals with higher levels of short video use are more likely to exhibit IA during functional tasks that require sustained engagement [17]. At the level of neural mechanisms, short video viewing activates the brain’s default mode network, which interacts with visual and auditory processing pathways while showing weaker connectivity with regions such as the prefrontal cortex that are responsible for attentional regulation and cognitive control [31]. This pattern suggests that sensory-driven processing predominates during short-video consumption, leading to impaired attentional control. Resting-state EEG studies further corroborate that individuals with stronger tendencies toward SVA demonstrate poorer attentional functioning [32]. The I-PACE (Interaction of Person-Affect-Cognition-Execution) model posits that the development of problematic internet use is attributable to a collective contribution of personal traits, emotional and cognitive responses, and individual executive function factors [33]. Within this theoretical framework, SVA, as an outcome of internet use disorder, has been demonstrated to exert a feedback effect on an individual’s cognitive responses, thereby influencing emotional reactions [34]. Firstly, IA falls under the Cognition component of the model. According to the model’s feedback mechanism, SVA affects an individual’s cognitive function. The distinctive attributes of short video content can readily prompt recurrent fluctuations in an individual’s focus, culminating in the manifestation of IA at the cognitive tier. Secondly, the I-PACE model underscores the interplay between cognition and affect. The presence of IA has been demonstrated to result in impaired regulation of negative stimuli and negative thoughts, thereby engendering difficulties in disengaging from negative thinking and information [35]. This has been demonstrated to engender an increased vulnerability to adverse emotional responses, such as anxiety and depression [36]. Consequently, within this process, IA may function as a pivotal mediating factor, thereby connecting SVA to negative emotions.

Existing research has provided preliminary evidence for an association between SVA and negative emotions. However, most studies have adopted a variable-centered approach, which may obscure within-group heterogeneity and limit the accurate identification of high-risk subgroups. In addition, although prior work has explored the relationships among IA, SVA, and negative emotions, the role of IA as a core cognitive factor in the pathway through which SVA influences negative emotions has not yet been empirically examined. In order to address these research gaps, the present study aims to achieve two core objectives: Firstly, employing a person-centered approach, it utilizes Latent Profile Analysis (LPA) to identify distinct categories of SVA among college students. This approach explores group heterogeneity that may be overlooked in previous studies. Secondly, by examining the mediating role of IA between SVA and negative emotions, the investigation will proceed to examine the underlying associative mechanisms. Based on the above objectives and theoretical framework, the following three hypotheses are proposed:

**H1.**  *There was a significant correlation between SVA and negative emotions.*

**H2.**  *There are distinct latent profiles of SVA among college students.*

**H3.**  *IA mediates the relationship between SVA and negative emotions.*

## 2. Materials and Methods

### 2.1. Participants

Participants were recruited through convenience sampling from five full-time undergraduate institutions across three Chinese cities. The sample encompassed all academic years, from freshman to senior level, and included students from a variety of disciplinary backgrounds, including liberal arts, science, engineering, and medicine. The inclusion criteria for the study were as follows: (1) full-time undergraduate status, (2) age between 18 and 23 years, (3) proficiency in smartphone operation. The following criteria were used to determine exclusion from the study: (1) Self-reported severe mental illness or recent major life stressors. (2) Completion time under 150 s. (3) Discernible patterned responses, such as selecting the same option for all questions. Initially, 435 questionnaires were disseminated. Following the collection phase, two researchers meticulously reviewed the data. In the end, 405 questionnaires were retained, which corresponds to a validity rate of 93.10%. According to the extant literature on the subject, the minimum sample size required for LPA to ensure result stability is typically 300 [37]. The 405 samples utilized in this study satisfied the requisite analytical standards.

### 2.2. Procedure

The present study scrupulously complies with the pertinent ethical principles stipulated in the Declaration of Helsinki and has been endorsed by the Ethics Committee of Xi’an Jiaotong University Health Science Center (Ethics Approval Number: 2022-1529). The research was conducted using a questionnaire survey method. During the questionnaire development phase, all measurement tools employed in the study were integrated into the “Wenjuanxing” online survey platform.

To facilitate participant recruitment, researchers contacted faculty members or class representatives at target universities and disseminated recruitment information through WeChat class groups. Following the dissemination of the recruitment notice, eligible students had the option of voluntarily participating in the study. No random selection or targeted screening was conducted. The recruitment announcement explicitly provided the link to the questionnaire and the QR code. Upon scanning the QR code or clicking the link to access the questionnaire homepage, participants were first presented with an informed consent form. This document meticulously delineates the research objectives, the voluntary nature of participation, the principle of anonymity, and the commitment that research data will be used solely for scientific purposes. Participants will only be permitted to proceed to the formal questionnaire response stage once they have checked the box indicating their consent.

The formal investigation was conducted from January to August of 2025. Participants are instructed to complete the questionnaire independently, with an average response time of approximately six minutes. Throughout the study, no personally identifiable information such as names or student ID numbers was collected, ensuring maximum participant privacy and enhancing the authenticity and reliability of responses. With regard to the implementation of quality control measures, the system utilized IP address restriction, thereby ensuring that each device was constrained to prevent multiple submissions and thus precluding the occurrence of duplicate responses. Furthermore, the research team meticulously excluded invalid samples with exceedingly brief response times during the subsequent phases, thereby ensuring the reliability of the research sample.

### 2.3. Measures

#### 2.3.1. General Information Questionnaire

A self-administered questionnaire was developed to collect socio-demographic information, including major category, gender, and residential location, as well as frequently used short video apps.

#### 2.3.2. Short Video Addiction

SVA was assessed using the revised Chinese Internet Addiction Scale (CIAS-R) [38]. The scale consists of 19 items across four dimensions: Compulsive Internet Access and Internet Addiction Withdrawal Reactions (Sym-C & Sym-W), and Tolerance Symptoms of Internet Addiction (Sym-T). Interpersonal and Health-Related Problems (RP-IH) and Time Management Problems (RP-TM). A score greater than 53 is classified as the addiction group. The phenomenon of SVA has been identified as a distinct manifestation of Internet addiction, exhibiting analogous characteristics, including compulsive utilization and withdrawal responses [39]. Consequently, the CIAS-R was selected for the study to assess SVA. During scale administration, the instructions explicitly asked participants to complete all items based on their short video use. The wording of individual items was not modified, thereby preserving fidelity to the original conceptualization of the CIAS-R. The CIAS-R has demonstrated high reliability in previous studies (Cronbach’s α = 0.970) [40]. In the present study, the Cronbach’s α was 0.934.

Given the adaptation of the instructions, Confirmatory Factor Analysis (CFA) was performed to ensure the scale’s structural validity in the context of SVA. The model exhibited an acceptable fit to the data: The four-factor model exhibited an acceptable fit: *χ*^2^(146) = 336.8, *p* < 0.001, *χ*^2^/*df* =2.307. The Comparative Fit Index (CFI) and the Tucker–Lewis Index (TLI) were 0.920 and 0.900, respectively. The Root Mean Square Error of Approximation (RMSEA) was 0.060 (90% CI [0.053, 0.070]), and the Standardized Root Mean Square Residual (SRMR) was 0.050. Furthermore, the measurement model demonstrated strong convergent validity, with all standardized factor loadings >0.50, Composite Reliability (CR) > 0.70, and Average Variance Extracted (AVE) > 0.50. These results support the structural applicability of the CIAS-R for assessing short video addiction in the present sample.

#### 2.3.3. Inattention

The present study employed the Inattention Subscale of the Adult ADHD Self-Report Scale (ASRS) in order to assess individuals’ levels of inattention (IA). The present scale was developed by Kessler et al. [41]. on the basis of DSM diagnostic criteria. The original scale comprises two dimensions, namely inattention and hyperactivity-impulsivity, with each dimension comprising nine items. Given the study’s emphasis on cognitive-level attention functioning, the Attention Deficit subscale was utilized for measurement. It has been demonstrated by preceding studies that this subscale functions as an autonomous and efficacious instrument for the evaluation of attention difficulties within the demographic of college students [42,43]. The items are scored on a 5-point Likert scale, with higher total scores indicating more severe IA. In the present study sample, this subscale demonstrated good internal consistency with a Cronbach’s α coefficient of 0.894.

#### 2.3.4. Negative Emotions

The present study employed the Hospital Anxiety and Depression Scale (HADS) to assess individuals’ negative emotions. The scale comprises 14 items, which are divided into two independent subscales: The anxiety (HADS-A) and depression (HADS-D) scales each comprise 7 items [44]. The items are scored using a 4-point Likert scale. It is important to note that higher scores on each subscale are indicative of more severe symptoms of anxiety or depression. Although originally designed for the hospital setting, the scale has been shown to demonstrate equivalent reliability and validity in a variety of non-clinical populations, including general community residents and college students [45,46]. The HADS was selected for this study due to its brevity, ease of administration, primary focus on emotional symptoms as opposed to somatic manifestations, and its efficacy in identifying anxiety and depressive tendencies that are prevalent among college students. Cronbach’s α in the current study was 0.833.

### 2.4. Data Analysis

The data were analyzed using IBM SPSS Statistics version 25.0, Mplus version 8.3, and JASP version 0.18.2. A significance level of α = 0.05 was established on both sides. A Harman’s single-factor test was used to identify potential common methodological biases [47].

Initially, a comprehensive set of descriptive statistics was conducted to ascertain the prevalence of SVA, IA, negative emotions, and general information. Continuous variables were expressed as mean ± SD, and categorical variables were expressed as frequencies and proportions. Pearson correlation analysis was used to test the correlation between SVA and individual dimensions, IA, depression, and anxiety.

Subsequently, latent profile analysis was conducted using Mplus version 8.3 to identify SVA profiles in college students. The optimal model was selected based on several fit indices. The Akaike Information Criterion (AIC), Bayesian Information Criterion (BIC), and adjusted Bayesian Information Criterion (aBIC) were used to compare the models, with lower values indicating a better fit. The entropy value ranges from 0 to 1, with a value closer to 1 indicating a more accurate classification. In addition, the *p* values of the Lo–Mendell–Rubin adjusted likelihood ratio test (LMR) and bootstrap likelihood ratio test (BLRT) were statistically significant, indicating that the k classification was a better-fitting model than the k − 1 classification [48]. After determining the optimal model, further analyses were performed using IBM SPSS Statistics version 25.0.

Finally, mediation effect analyses were conducted on the multi-category independent variables using Mplus version 8.3, as suggested in the references [49]. Relative mediation analyses were conducted by selecting the potential profile of SVA as a reference for relative mediation effects, direct effects, and total effects. The assessment of relative mediation effect significance was also grounded in the Bootstrap method.

## 3. Results

### 3.1. Characteristics of the Sample

The characteristics of the 405 college students are shown in Table 1. The distribution of students across different fields of study was relatively balanced, with 44.0% *(n* = 178) being medical students. The predominant gender was female (72.6%). The majority of the students (54.1%) were from urban areas. The most frequently used short video app was Douyin (61.2%).

### 3.2. Common Method Variance Test

The present study employed a self-assessment questionnaire, a method which is susceptible to common methodological biases. Consequently, Harman’s one-way test was conducted to ascertain the potential presence of common methodological biases. An exploratory factor analysis was subsequently conducted on the entire questionnaire used in this study, with factors being extracted using the principal components approach. The results indicated that 7 factors exhibited an eigenroot greater than one, with the first factor accounting for 30.21% of the variance (less than the critical value of 40%). This finding suggests that there was no substantial issue of common method bias in this study.

### 3.3. Correlation Analysis of SVA with IA, Anxiety and Depression

**Figure 1:**
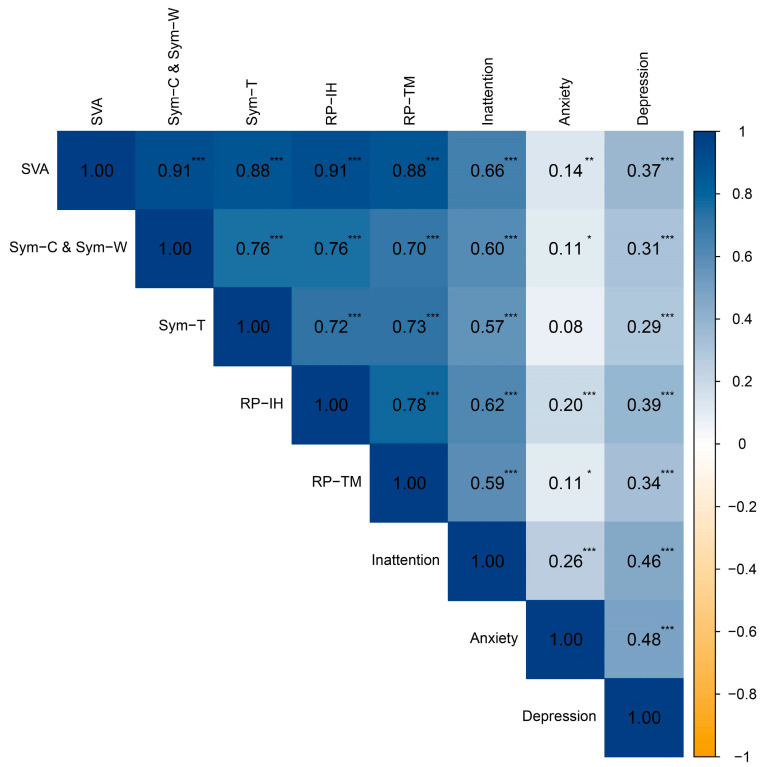
Correlation analysis of SVA with IA, anxiety and depression. * *p* < 0.05, ** *p* < 0.01, *** *p* < 0.001.

As Figure 1 shows, the present study found a positive correlation between SVA and IA, Anxiety, and Depression. Furthermore, a positive correlation was also identified between IA and Anxiety and Depression. Concurrently, all dimensions of SVA exhibited a positive correlation with IA, Anxiety, and Depression, with the exception of the Tolerance Symptoms of Internet Addiction dimension.

### 3.4. Latent Profile Analysis of SVA

The fitting information of the LPA model of SVA is shown in Table 2. As the number of profiles increases, AIC, BIC, and aBIC gradually decrease, while entropy consistently remains above 0.80. For BLRT, the results show a statistically significant enhancement in fit with each supplementary profile added to the model (*p* < 0.001). However, for LMR, only the 2-class and 3-class models reached statistical significance, indicating that both were prioritized and that the 3-class model was more accurate than the 2-class model. By combining these tests, the 3-class model was chosen as the final model.

**Figure 2:**
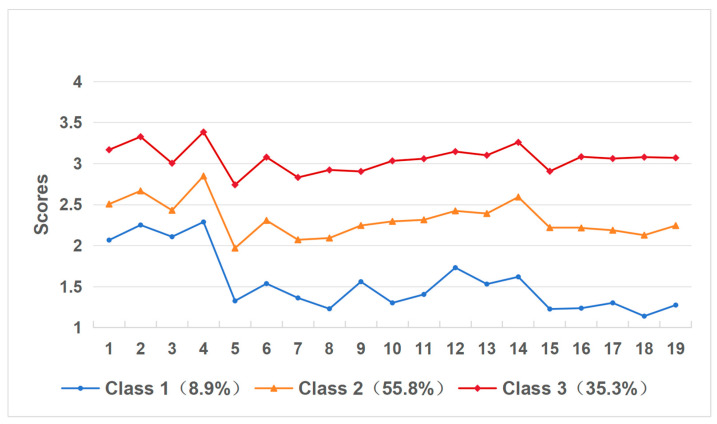
Patterns for three distinct profiles.

The profiles of the three potential categories of college students’ SVA are illustrated in Figure 2. Class 1 (*n* = 36, 8.9%), characterized by the lowest scores on all SVA symptom dimensions, was designated “Healthy short video use”. Class 2 (*n* = 226, 55.8%), who scored at an intermediate level on all dimensions, significantly higher than Class 1 but lower than Class 3, had formed a habit of dependence with a high risk of addiction and were named “Short video dependence”. Class 3 (*n* = 143, 35.3%) demonstrated the highest mean scores across all SVA dimensions. The mean CIAS score for this group was 58.217 ± 5.603, which exceeds the established clinical cutoff of 53 for problematic internet use. This finding indicates that individuals within this subgroup exhibit symptoms of clinically significant addiction that surpass those observed in other characteristic groups. Thus, it was named “Short video addiction”.

### 3.5. Differences in SVA, IA, Anxiety, and Depression Across Latent Profiles

As illustrated in Table 3, a clear distinction emerges among the three distinct groups with respect to their scores on each measure. The findings of the latent profile analysis demonstrated that the three groups exhibited significant disparities on the variables of SVA, IA, anxiety, and depression. Healthy short video group use had the lowest scores. Short video addiction group had the highest scores. Short video dependence group fell in the middle.

### 3.6. Relative Mediation Analysis Between SVA Profiles

**Figure 3:**
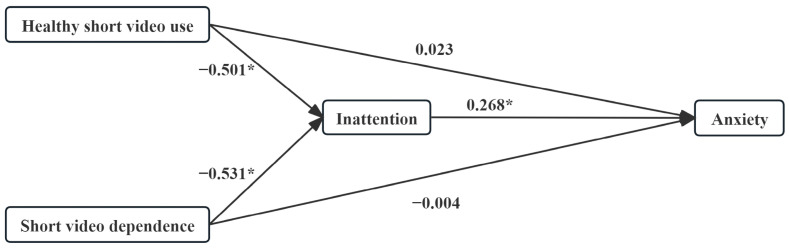
Relative mediation modeling of SVA and Anxiety. Short video addiction was used as a reference. * *p* < 0.05.

In the anxiety model, with Short video addiction as the reference, the relative total effects of Healthy short video use and Short video dependence were *β* = −0.112 (*p* = 0.166) and *β* = −0.146 (*p* = 0.001), respectively. Of these, only Short video dependence reached statistical significance. The direct effect was not significant for either group (*β* = 0.023, *p* = 0.799; *β* = −0.004, *p* = 0.956). IA was a significant positive predictor of anxiety (*β* = 0.268, *p* = 0.003). The 95% CI for the relative mediated effects of Healthy short video use and Short video dependence were [−0.222, −0.041] and [−0.239, −0.039], respectively. Neither contained zero, suggesting that IA played a significant mediating role in both, while IA fully mediated in Short video dependence, with relative mediating effects of *β* = −0.134 and *β* = −0.142, respectively (Table 4, Figure 3).

**Figure 4:**
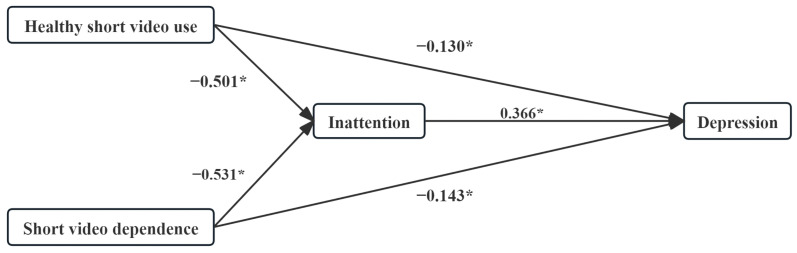
Relative mediation modeling of SVA and Depression. Short video addiction was used as a reference. * *p* < 0.05.

Within the framework of the depression model, after controlling for potential confounding variables and employing Short video addiction as a point of reference, both the relative total effect (*β* = −0.314, *p* < 0.001; *β* = −0.337, *p* < 0.001) and the relative direct effect (*β* = −0.130, *p* = 0.040; *β* = −0.143, *p* = 0.006) were found to be statistically significant. IA was a significant positive predictor of depression (*β* = 0.366, *p* < 0.001). The 95% CI for the relative mediated effects of Healthy short video use and Short video dependence were [−0.258, −0.122] and [−0.265, −0.129], respectively. It was evident that neither variable overlapped with a null value of 0.00, thereby indicating that IA exhibited a statistically significant relative mediation effect in both cases. The relative mediation effects were *β* = −0.184 for Healthy short video use and *β* = −0.195 for Short video dependence, respectively (see Table 4 & Figure 4).

## 4. Discussion

The study revealed that only a negligible proportion of the sampled college students exhibited Healthy short video use behaviors, with a percentage of 8.9% being identified as demonstrating such practices. In addition, the study identified three SVA characteristics among a sample of college students: namely, Healthy short video use, Short video dependence, and Short video addiction. IA was found to mediate the association between SVA and anxiety and depression, and to have a fully mediating role between SVA and anxiety.

First, consistent with previous research from diverse regions [50,51,52], the current study found a positive relationship between SVA and negative emotions, supporting Hypothesis 1. The usage and content characteristics of short video platforms may play an important role in this relationship. It is suggested that college students, as the primary demographic of short video apps, are more susceptible to the influence of “upward comparison” [53]. The prevalence of curated, idealized life clips on short video platforms can engender unconscious social comparisons among college students, exacerbating feelings of inadequacy. Excessive viewing has been demonstrated to trigger negative emotions such as anxiety and depression [54,55]. Concurrently, the utilization of algorithms to recommend short video is poised to further consolidate this process. Intelligent recommendation mechanisms expose users to a single, stable form of content, forming an “information cocoon”. Consequently, it has been demonstrated that this phenomenon increases the likelihood of exposure to content that induces comparisons and limits the individual’s access to new information [56]. Despite the variability in usage patterns across regions attributable to technological differences, extant studies consistently indicate that prolonged exposure to highly repetitive short video content leads to decreased satisfaction among college students, accompanied by increased feelings of boredom, irritability, and depression [57,58,59]. Furthermore, addiction to the use of short videos can consume students’ time and opportunities for realistic social interactions, thereby engendering a deficiency in the realm of emotional well-being and social support [60]. However, such deficiencies may lead to social maladjustment and heightened feelings of loneliness, exacerbating anxiety and depression [61].

The findings indicate heterogeneity in short video application use among the college students included in this study and that distinct risk stratifications are present. This finding is consistent with Hypothesis 2. Latent profile analysis results indicate significant heterogeneity in the sampled college students’ short video usage behavior, with the optimal model identifying three latent subtypes: Healthy short video use (8.9%), Short video dependence (55.8%), and Short video addiction (35.3%). This finding aligns with existing research on risk stratification for SVA [54]. The study indicates that the Healthy short video use group exhibits the lowest percentage, while the Short video dependence group demonstrates the highest percentage, suggesting that a substantial proportion of the sampled students are at high risk of SVA, despite not having developed SVA symptoms. The phenomenon of the Short video dependence group is of particular concern, as they are in a ‘critical state’: their short video use behaviors are not consistent with healthy patterns, but are still reversible. The emergence of such groups appears to be associated with the immediate gratification derived from short videos and the individuals’ limited capacity for emotional regulation. Research suggests that some individuals consume short video content to momentarily alleviate pressures from their daily lives and regulate their emotional states [62]. However, the pleasurable experience derived from this external stimulus is fleeting and unstable. When the intensity of the stimulus diminishes or usage is interrupted, negative emotions are more likely to emerge [63]. In this process, individuals may gradually increase the frequency or duration of short video usage to regain pleasurable experiences and alleviate negative emotions [64]. This reinforces repetitive behavior, promotes dependency, and may even develop into addiction. Consequently, extant research findings indicate that broadening the scope of prevention and control of SVA to encompass groups that have not yet developed addiction may yield more efficacious outcomes. For instance, the prioritization of early identification and intervention for this group of dependent users, who constitute a significant proportion, is imperative. Concurrently, intervention strategies should not be confined to encouraging students to curtail their utilization of short video applications. Educational institutions and pedagogues may assume a pivotal function in assisting students in recognizing patterns of excessive short video usage. Furthermore, educational institutions and pedagogues are encouraged to promote student participation in offline activities, such as physical exercise and social clubs, thereby replacing the transient gratification derived from the consumption of audiovisual media with authentic achievements [65,66]. Guiding students to systematically plan fragmented time and enhance self-management skills will help them break free from dependence on short videos, effectively preventing SVA.

The results indicated that demographic variables, including Major Category, gender, residential location, and frequently used short video apps, did not differ significantly across SVA risk groups. At present, smartphone use has become highly prevalent, and short video applications, due to their immediacy and low access threshold, are widely used for emotion regulation and compensatory social interaction [67]. Within the context of the present sample, short video use behaviors may be broadly distributed across individuals with diverse demographic backgrounds, thereby diminishing the discriminatory value of traditional demographic variables in risk stratification. The findings suggest that screening for high-risk individuals may overlook a large number of potentially dependent or addicted individuals if only demographic information is used, and that colleges and universities can pay more attention to students’ psychological states and motivations for use in their screening and prevention efforts.

In accordance with Hypothesis 3, the findings suggest that IA significantly mediated the relationship between SVA and negative emotions, though its mediating pattern differed across emotional outcomes. Specifically, IA fully mediated the relationship between SVA and anxiety, while it partially mediated the relationship between SVA and depression. This finding indicates that the mechanisms through which SVA affects various negative emotions may not be entirely uniform. In the anxiety model, IA exhibited full mediation, suggesting that the association between SVA and anxiety may be indirect and primarily operate through IA. Individuals experiencing anxiety frequently encounter difficulties in maintaining their attention. They often become excessively engrossed in uncertain or negative information, thereby exacerbating feelings of tension and worry. Excessive consumption of short videos has the potential to diminish an individual’s capacity for goal-directed attention, as it diverts attentional resources [68]. This can impede the ability to disengage from negative information and ruminative thoughts [69]. Therefore, the cognitive load imposed by SVA translates into persistent tension and anxiety. It is possible that college students are perpetually immersed in an environment characterized by substantial academic workloads and multifarious learning demands. This persistent cognitive load renders them more susceptible to manifesting symptoms of IA. Persistent IA has been shown to readily transform into doubts and worries about task completion and self-efficacy, thereby triggering anxiety. Consequently, for college students with higher levels of short video addiction, IA is not only a behavioral manifestation but also likely serves as a significant cognitive pathway for anxiety generation.

In the depression model, IA shows partial mediating effects. This suggests that IA may not be the primary pathway through which SVA is associated with depression. Anxiety is marked by an immediate attentional bias and generally appears as easily distracted attention. In contrast, depressive mood forms over time and involves longer-lasting changes in thought, emotion, and behavior. The high-frequency stimulation and fast-paced nature of short videos require individuals to engage in frequent attentional switching [70]. Prolonged exposure to such distraction can readily trigger IA. For college students, IA can lead to decreased learning efficiency, poor academic performance, and unattainable goals, resulting in a sense of powerlessness and self-denial. When this frustrating experience builds up, depression becomes more likely [71]. However, cognitive deficits such as IA alone cannot fully explain the association between SVA and depression. Existing evidence indicates that excessive short-video consumption displaces time from physical activity and face-to-face social interaction, resulting in reduced social-emotional support and insufficient physical activity [72,73], both of which are well-established risk factors for depression. These results indicate that SVA contributes to depressive mood through both IA and behavioral or social factors. IA constitutes only one of the pathways involved.

Therefore, colleges and universities can actively identify students with short video dependence and addiction and offer psychological counseling to assist them in mitigating adverse emotional responses. In the case of anxiety, a potential intervention target is to enhance students’ capacity for effective attention management. This is achieved through interventions such as attention control training and the promotion of balanced scheduling of rest and recreation periods. In the case of depression, the interventions required must be more comprehensive in nature. This should encompass attention training, as well as the organization of regular social activities and mental health education. The purpose of this is to reduce feelings of loneliness and negative thinking.

There are several limitations to this study. First, the present study employed convenience sampling, a method that may limit the generalizability of its conclusions. Given that the participants were drawn exclusively from several universities in a specific region, the findings may not fully represent college students from diverse geographical and cultural backgrounds. Future research should utilize random sampling or multi-center studies to further validate these results. Second, due to the cross-sectional survey design, it was not possible to determine causal relationships between SVA, IA, and negative emotions. Third, the study data were derived from a self-reporting program, which may be subject to social desirability bias and recall inaccuracies. Future research should consider incorporating objective behavioral data to mitigate these biases. Finally, the covariates incorporated into this study were limited. Important factors such as academic workload and prior clinical diagnoses, which might be associated with the core variables not included in the observations. In order to ensure the generalizability of the findings, the research team will focus on conducting longitudinal studies and expanding the population and geographic coverage of the survey in the future.

## 5. Conclusions

The present study contributes to the extant literature by examining the relationship between SVA and negative emotions among college students. The findings of the present study reveal that higher SVA is associated with an increased risk of negative emotions. Furthermore, IA fully mediated the specific pathway from SVA to anxiety. Consequently, educational institutions and pedagogues must expeditiously ascertain students’ SVA and furnish assistance to ameliorate attentional attrition and the advent of negative emotions.

## Figures and Tables

**Table 1:** Demographic information on survey respondents (*n* [%]).

Variables	Category	Number	Class 1 (*n* = 36)	Class 2 (*n* = 226)	Class 3 (*n* = 143)	*χ* ^2^
Major Category	Science & Engineering students	127 (31.3%)	14 (38.9%)	68 (30.0%)	45 (31.5%)	2.674
	Humanities & Social Sciences students	100 (24.7%)	7 (19.4%)	53 (23.5%)	40 (28.0%)	
	Medical students	178 (44.0%)	15 (41.7%)	105 (46.5%)	58 (40.5%)	
Gender	Male	111 (27.4%)	8 (22.2%)	63 (27.9%)	40 (28.0%)	0.534
	Female	294 (72.6%)	28 (77.8%)	163 (72.1%)	103 (72.0%)	
Residential location	Urban	219 (54.1%)	19 (52.8%)	128 (56.6%)	72 (50.3%)	1.421
	Rural	186 (45.9%)	17 (47.2%)	98 (43.4%)	71 (49.7%)	
App	Bilibili	55 (13.6%)	3 (8.3%)	34 (15.0%)	18 (12.6%)	2.110
	Douyin	248 (61.2%)	22 (61.1%)	137 (60.6%)	89 (62.2%)	
	Kuaishou	21 (5.2%)	3 (8.3%)	11 (4.9%)	7 (4.9%)	
	Xiaohongshu	81 (20.0%)	8 (22.2%)	44 (19.5%)	29 (20.3%)	

**Table 2:** Fitting statistics and group size of latent profile analysis.

Number	AIC	BIC	aBIC	Entropy	LMR	BLRT	Profile Size (%)
1	17,981.198	18,133.346	18,012.767	—	—	—	—
2	15,919.834	16,152.059	15,968.018	0.906	<0.001	<0.001	55.6/44.4
3	15,250.009	15,562.313	15,314.809	0.944	<0.001	<0.001	8.9/55.8/35.3
4	14,912.267	15,304.648	14,993.682	0.931	0.118	<0.001	8.6/48.6/36.0/6.8
5	14,746.820	15,219.279	14,844.850	0.897	0.181	<0.001	6.9/33.1/28.6/26.9/4.4

Abbreviations: AIC, Akaike Information Criterion; BIC, Bayesian Information Criterion; aBIC, adjusted Bayesian Information Criterion; LMR, Lo–Mendell–Rubin adjusted likelihood ratio test; BLRT, bootstrap likelihood ratio test.

**Table 3:** Differences in SVA, IA, Anxiety, and Depression Across Latent Profiles.

Variable	Class 1 M (SD)	Class 2 M (SD)	Class 3 M (SD)	F	Post Hoc Test ^a^
SVA	29.361 ± 5.271	44.133 ± 4.341	58.217 ± 5.603	640.083 ***	1 < 2 < 3
IA	11.139 ± 6.481	15.544 ± 4.442	22.336 ± 5.801	106.515 ***	1 < 2 < 3
Anxiety	6.472 ± 5.609	6.810 ± 3.236	7.811 ± 2.821	4.605 *	2 < 3
Depression	6.806 ± 4.139	8.190 ± 3.057	10.406 ± 2.678	32.135 ***	1 < 2 < 3

Note: ^a^ *p* < 0.05, Bonferroni corrected. * *p* < 0.05, *** *p* < 0.001.

**Table 4:** The mediating effect of IA on SVA and negative emotions in college students.

Paths ^a^	*β*	SE	*p*	LLCI	ULCI
Inattention					
Class 1 (a1)	−0.501	0.056	<0.001	−0.604	−0.387
Class 2 (a2)	−0.531	0.037	<0.001	−0.596	−0.452
Anxiety (Direct effects)					
Inattention (b)	0.268	0.092	0.003	0.074	0.432
Class 1 (c1)	0.023	0.088	0.799	−0.151	0.191
Class 2 (c2)	−0.004	0.068	0.956	−0.145	0.124
Indirect effects					
a1 × b	−0.134	0.047	0.004	−0.222	−0.041
a2 × b	−0.142	0.052	0.006	−0.239	−0.039
Total effects					
a1 × b + c1	−0.112	0.081	0.166	—	—
a2 × b + c2	−0.146	0.045	0.001	—	—
Inattention					
Class 1 (a1)	−0.501	0.056	<0.001	−0.604	−0.387
Class 2 (a2)	−0.531	0.037	<0.001	−0.596	−0.452
Depression (Direct effects)					
Inattention (b’)	0.366	0.057	<0.001	0.247	0.471
Class 1 (c1’)	−0.130	0.063	0.040	−0.260	−0.012
Class 2 (c2’)	−0.143	0.052	0.006	−0.242	−0.040
Indirect effects					
a1 × b’	−0.184	0.035	<0.001	−0.258	−0.122
a2 × b’	−0.195	0.034	<0.001	−0.265	−0.129
Total effects					
a1 × b’ + c1’	−0.314	0.062	<0.001	—	—
a2 × b’ + c2’	−0.337	0.043	<0.001	—	—

Abbreviations: *β*, linear regression beta coefficients; LLCI, 95% confidence interval lower; ULCI, 95% confidence interval upper; SE, standard error. Note: ^a^ “Short video addiction” as the reference group.

## Data Availability

The data presented in this study are available on request from the corresponding author. The data are not publicly available due to ethical restrictions and participant privacy.

## Abbreviations

The following abbreviations are used in this manuscript:

SVA	Short video addiction
IA	Inattention
LPA	Latent profile analysis
Sym-C & Sym-W	Compulsive Internet Access and Internet Addiction Withdrawal Reactions
Sym-T	Tolerance Symptoms of Internet Addiction
RP-IH	Health-Related Problems
RP-TM	Time Management Problems
